# Clinical Value of Machine Learning-Based Ultrasomics in Preoperative Differentiation Between Hepatocellular Carcinoma and Intrahepatic Cholangiocarcinoma: A Multicenter Study

**DOI:** 10.3389/fonc.2021.749137

**Published:** 2021-11-05

**Authors:** Shanshan Ren, Qian Li, Shunhua Liu, Qinghua Qi, Shaobo Duan, Bing Mao, Xin Li, Yuejin Wu, Lianzhong Zhang

**Affiliations:** ^1^ Henan University People’s Hospital, Zhengzhou, China; ^2^ Henan Provincial People’s Hospital, Zhengzhou, China; ^3^ Henan Provincial Cancer Hospital, Zhengzhou, China; ^4^ First Affiliated Hospital of Zhengzhou University, Zhengzhou, China

**Keywords:** hepatocellular carcinoma, intrahepatic cholangiocarcinoma, machine learning, radiomics, ultrasonography

## Abstract

**Objective:**

This study aims to explore the clinical value of machine learning-based ultrasomics in the preoperative noninvasive differentiation between hepatocellular carcinoma (HCC) and intrahepatic cholangiocarcinoma (ICC).

**Methods:**

The clinical data and ultrasonic images of 226 patients from three hospitals were retrospectively collected and divided into training set (*n* = 149), test set (*n* = 38), and independent validation set (*n* = 39). Manual segmentation of tumor lesion was performed with ITK-SNAP, the ultrasomics features were extracted by the pyradiomics, and ultrasomics signatures were generated using variance filtering and lasso regression. The prediction models for preoperative differentiation between HCC and ICC were established by using support vector machine (SVM). The performance of the three models was evaluated by the area under curve (AUC), sensitivity, specificity, and accuracy.

**Results:**

The ultrasomics signatures extracted from the grayscale ultrasound images could successfully differentiate between HCC and ICC (*p* < 0.05). The combined model had a better performance than either the clinical model or the ultrasomics model. In addition to stability, the combined model also had a stronger generalization ability (*p* < 0.05). The AUC (along with 95% CI), sensitivity, specificity, and accuracy of the combined model on the test set and the independent validation set were 0.936 (0.806–0.989), 0.900, 0.857, 0.868, and 0.874 (0.733–0.961), 0.889, 0.867, and 0.872, respectively.

**Conclusion:**

The ultrasomics signatures could facilitate the preoperative noninvasive differentiation between HCC and ICC. The combined model integrating ultrasomics signatures and clinical features had a higher clinical value and a stronger generalization ability.

## Introduction

Primary liver cancer (PLC) is the second most common cause of cancer-related death worldwide (1, 2). The incidence and mortality of PLC are steadily increasing (3), which is a great threat to global public health. Histologically, PLC is divided into hepatocellular carcinoma (HCC), intrahepatic cholangiocarcinoma (ICC), and rare types (less than 1%), such as mixed liver cancer (4). Although HCC and ICC share some similar risk factors and clinical manifestations, they differ in molecular features and carcinogenic mechanism (5). Therefore, the therapeutic decision-making and the prognosis also differ between the two (6). For HCC patients, surgical resection remains the first-line treatment (7). Early ICC is usually asymptomatic and the appearance of clinical symptoms may indicate the spread and metastasis of cancer. ICC generally remains undetected until the late stage. These features of ICC have limited the choices of surgery or liver transplantation for ICC patients. According to international guidelines, accurate differentiation between HCC and ICC is a prerequisite for sufficient first-line therapy of patients (8–10). Also, the survival and the prognosis of ICC patients are usually worse than those of HCC patients (11). It is critical to differentiate between HCC and ICC before surgery to make correct clinical decisions and prognostic predictions.

Generally, HCC and ICC are diagnosed based on imaging and serological and pathological evaluations (12). It was realized that the naked eye could identify limited information, and the conventional preoperative imaging evaluation could be highly subjective and differed based on the radiologist’s experience. It may fail to detect hidden metastases or determine the infiltration scope of the tumor lesions (13). Also, in patients with liver cirrhosis, the conventional imaging techniques can hardly differentiate between small lesions of ICC and HCC. This is because most ICC and HCC lesions share a similar enhancement pattern (14). Given the facts above, the conventional imaging techniques only have a limited application value in tumor patients. Alpha-fetoprotein (AFP) and carbohydrate antigen 19-9 (CA19-9) are considered the ideal serum tumor markers for HCC and ICC. However, these two tumor markers are generally unsatisfactory in diagnostic sensitivity or specificity. They may be unreliable if the diagnosis of tumors is made based on them alone (15, 16). As the risk of cancers increases at the late stage, tumor biopsy does not apply in most situations (17). At present, it is urgent to look for a preoperative noninvasive method to differentiate between HCC and ICC.

Radiomics is an emerging technology, which deals with the extraction of a large bulk of information from medical images with high throughput, for example, shape, grayscale, textures, and wavelets. Radiomics involves deeper mining, prediction, and analysis of the extracted information to make more accurate diagnoses and tap into the full potential of medical imaging. In recent years, radiomics has been widely applied to tumor diagnosis (18–20), pathology grade (21, 22), vascular invasion and therapeutic evaluation (23, 24), and prognostic prediction (25, 26). Compared with other imaging techniques, ultrasound has the advantages of low cost, easy operation, immediate result interpretation after examination, and no radiation exposure (27, 28). Due to these advantages, the clinical application of ultrasomics is worthy of further investigation. Ultrasomics has been proven useful in the early diagnosis, preoperative grading prediction, efficacy evaluation and prognosis evaluation of liver tumor, breast tumor, thyroid tumor, gastrointestinal tumor, glioma, and other common tumor diseases (29–32). However, there are few reports on the preoperative differentiation between HCC and ICC based on ultrasomics. Peng et al. applied ultrasomics to preoperative noninvasive differentiation between the histopathological subtypes of PLC (33). However, their study was confined to a single center and lacked of further validation of the findings. The present study was intended to investigate the clinical value of ultrasomics signatures in preoperative differentiation between HCC and ICC. The model performance was also tested on an independent validation set.

## Materials and Methods

### Study Population

**Figure 1:**
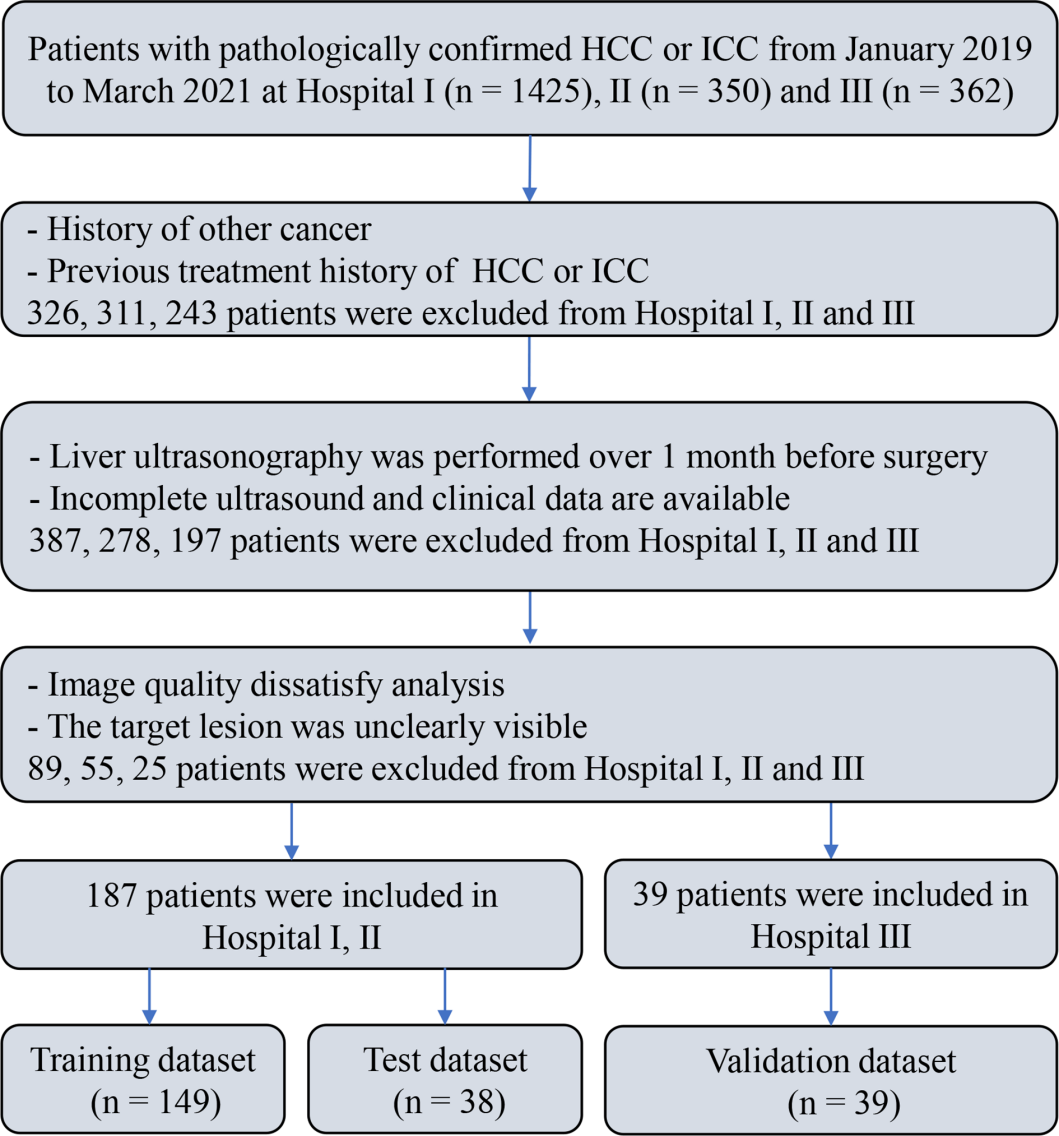
The flowchart of inclusion and exclusion of the study population.

A multicenter retrospective study involving three hospitals was performed, which was approved by the ethics committee. Informed consent was waived given the retrospective nature of the study. Clinical data and ultrasound images were collected from 2,137 patients pathologically confirmed as HCC or ICC at three hospitals from January 2019 to March 2021. Among them, 226 patients (HCC = 176, ICC = 50) were included in the final analysis. The inclusion criteria were as follows: (1) being pathologically confirmed as HCC or ICC; (2) having received liver ultrasound within 1 month before surgery and the ultrasound images information being intact; (3) having not received antitumor treatments before, including liver transplantation (LT), microwave ablation (MWA), radiofrequency ablation (RFA), and transcatheter arterial chemoembolization (TACE); (4) the ultrasound images satisfying the analytical requirements and the target lesions being totally visible on the ultrasound images; and (5) no history of concurrent malignancies. The flow chart of subject inclusion and exclusion is shown in **Figure 1**.

Patients from two hospitals (Henan Provincial Peoples Hospital and the First Affiliated Hospital of Zhengzhou University) (*n* = 187, HCC = 146, ICC = 41) were mixed and divided into training set (*n* = 149, HCC = 118, ICC = 31) and test set (*n* = 38, HCC = 28, ICC = 10) by stratified sampling at a ratio of 8:2. The patients from a third hospital (Henan Cancer Hospital) (*n* = 39, HCC = 30, ICC = 9) were used as an independent validation set.

### Clinicopathological Characteristics of Patients

The clinical data were acquired from the electronic health records, including: demographics (gender, age, history of hepatitis), laboratory tests (AFP, ALT, AST, TB, CB, and UCB) and ultrasound features (size of lesion). The laboratory tests and ultrasound imaging were examined within 1 month before surgery. The patients’ pathology information (the pathological diagnosis of HCC or ICC) was obtained from the pathology information system.

### Imaging Acquisition and Segmentation

Ultrasound images of liver tumors were collected using the Color Doppler Ultrasound System with a convex array transducer (frequency range 2.5–6 MHz), including GE Logiq E9, GE Vivid E9, HI VISION Ascendus, HI ALOK ProSound A5, Philips EPIQ 5, and Aloka EZU-MT28-S1. All ultrasound scans were performed by ultrasound physicians who had over 5 years of experience in liver ultrasound. At least one original ultrasound image showing the lesion and the same image containing the measurement parameters were stored in the DICOM format.

**Figure 2:**
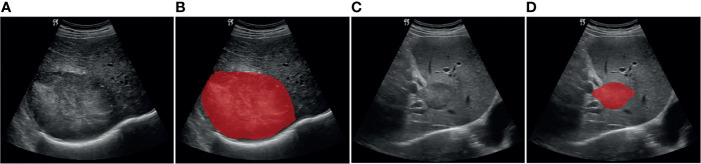
Example of delineating region of interest (ROI) on grayscale ultrasound images. **(A, B)** Patient with HCC. **(C, D)** Patient with ICC.

The open-source software ITK-SNAP v.3.6.0 was used to manually delineate the region of interest (ROI) (34). First, an ultrasound physician with over 9 years of experience loaded the images into the ITK-SNAP software and manually annotated the entire lesion. Another ultrasound physician with 30 years of experience then delineated ROI in the lesions for all ultrasound images. The reproducibility of feature extraction from ROI was evaluated according to the delineation results. Both ultrasound physicians had 4 years of working experience concerning ITK-SNAP software. They were blinded to clinical history and pathology results but were aware of the purpose and design of the study. The ROI segmentation results for the representative liver lesions are shown in **Figure 2**.

### Feature Extraction and Selection

A researcher with 5 years of experience performed image preprocessing to eliminate variability of the ultrasound images arising from the use of different ultrasound equipment at different hospitals and to improve the reproducibility of feature extraction. First, the ultrasound images were normalized based on the mean and standard deviation. Second, the images were resampled by B-spline interpolation to 1 mm × 1 mm pixel. Finally, gray-level discretization was performed for the histogram with the bin width fixed at 25 (35).

The open-source Python package Pyradiomics v.2.1.2 was used to extract ultrasound features from each patient. The extracted features were divided into the following seven categories (36): (I) first-order statistical features; (II) two-dimensional shape features; (III) gray-level cooccurrence matrix (GLCM); (IV) gray-level run length matrix (GLRLM); (V) gray-level size-zone matrix (GLSZM); (VI) gray-level dependence matrix (GLDM); (VII) neighborhood gray-tone-difference matrix (NGTDM). Fourteen filters were applied on the original images to obtain the derivative images for each patient. Except for the shape features, the features of all categories were directly obtained from the original and derivative images. Detailed information about the feature extraction method, filters, and features is available in the **Supplementary Materials 1, 2**.

**Figure 3:**
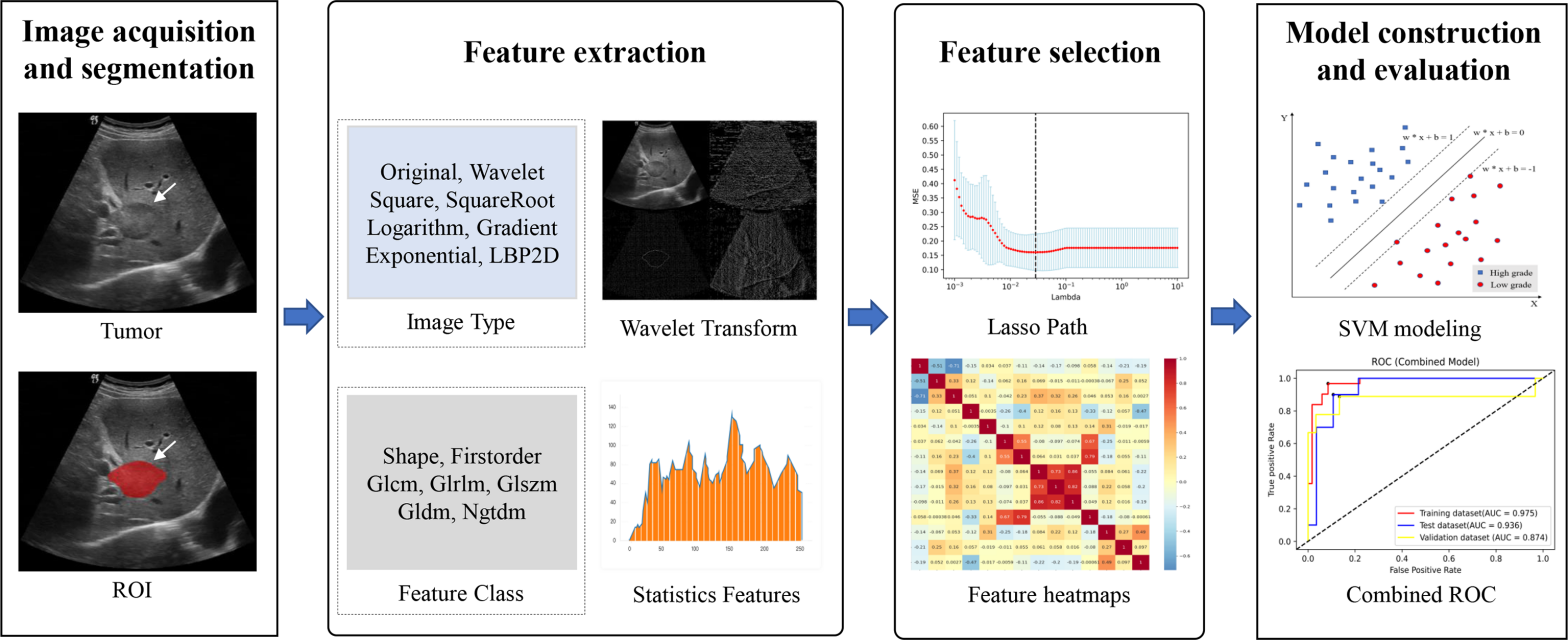
Overall flowchart of the study, including image acquisition and segmentation, feature extraction and feature selection, and model construction and evaluation.

Since the unit and value range varied for different extracted features, the feature values were of varying scales. To cope with this problem, we performed Z-score normalization before feature selection to ensure a relatively uniform distribution of the image features. However, all of the extracted features were high dimensional. The use of high-dimensional features might have the problems of low computational efficiency and overfitting (37). First, the features with zero variance were excluded by using the variance filtering method. Next, lasso method was performed for further dimensionality reduction of the features and the most valuable features were selected. The 10-fold cross-validation process was repeated 1,000,000 times to obtain the optimal value of parameter *λ*, which was introduced into the lasso method to calculate the regression coefficients of each feature. Finally, the features with nonzero coefficient were selected. The study workflow is shown in **Figure 3**.

### Machine Learning Model Construction and Evaluation

We invoked the Python scikit-learn 0.23.2 package for SVM model training and performance evaluation. The patients from two hospitals were randomly divided into training set and test set by stratified sampling at a ratio of 8:2. The patients from a third hospital were used as an independent validation set. The learning curve and the grid search were used concomitantly to select the optimized parameter combination consisting of the kernel function, coefficient of kernel function, penalty coefficient and *class_weight*. The specific process of parameter tuning is available in the **Supplementary Material 3**.

Three models were constructed in this paper. First, the clinical model was constructed using the patients’ clinical data, including gender, age, history of hepatitis, AFP, ALT, AST, TB, CB, UCB, and the size of lesion. Second, an ultrasomics model was constructed using the ultrasomics signatures extracted and selected from the ROI delineated on the ultrasound images of the HCC or ICC patients. Finally, the combined model was built by integrating the clinical features and the ultrasomics signatures. The details of the model construction process can be found in the **Supplementary Material 4**.

The three models built upon the training set were evaluated using the test set and the independent validation set. The predictive performance of the three models was evaluated by plotting the ROC and estimating the performance indicators, including AUC (along with 95% CI), accuracy, sensitivity, and specificity. An overview of the entire process is shown in **Figure 3**.

### Statistical Analysis

SPSS 25.0 software was used for statistical analysis. The normality of continuous variables was tested using the Kolmogorov-Smirnov test. Continuous variables obeying a normal distribution were analyzed by the independent-samples *t*-test. Otherwise, they were analyzed by Wilcoxon’s rank-sum test. The relationships between the categorical variables were tested by using the chi-square test. The continuous variables obeying a normal distribution were expressed as mean ± standard deviation. Otherwise, the continuous variables were expressed by medians [interquartile range (IQR)]. Categorical variables were expressed as *n* (%). *p* < 0.05 indicated a significant difference. The Delong test was employed for a quantitative comparison of the ROC among the three models (38).

The reproducibility of feature extraction was evaluated using the intraclass correlation coefficient, which greater than 0.8 indicated high consistency, 0.5 to 0.79 moderate consistency, and less than 0.5 low consistency (39).

## Results

### Clinicopathological Characteristics of Patients

The clinicopathological features in the training set, test set, and independent validation set are shown in **Table 1**. The percentages of ICC patients in the training set, test set, and independent validation set were 20.8% (31/149), 26.3% (10/38), and 23.1% (9/39), respectively. The percentages of ICC patients with a history of hepatitis were 63.8% (95/149), 73.7% (28/38), and 74.4% (29/39), respectively. The average age of patients was 57.2 ± 11.1, 58.7 ± 9.3, and 59.1 ± 11.2 in the training set, test set, and independent validation set, respectively. The three sets did not differ significantly in demographics, laboratory test results, and ultrasound features (*p* > 0.05).

**Table 1:** The clinicopathological features in the training set, test set, and validation set.

	Training set (*n* = 149)	Test set (*n* = 38)	*p-*value	Validation set (*n* = 39)	*p-*value
Gender			0.55		0.70
Male	105 (70.5%)	29 (76.3%)		29 (74.4%)	
Female	44 (29.5%)	9 (23.7%)		10 (25.6%)	
Age (years)^a^	57.2 ± 11.1	58.7 ± 9.3	0.43	59.1 ± 11.2	0.35
Liver diseases			0.34		0.26
Hepatitis	95 (63.8%)	28 (73.7%)		29 (74.4%)	
Other	54 (36.2%)	10 (26.3%)		10 (25.6%)	
AFP (ng/ml)^b^	13.5 (4.0–764.8)	7.2 (3.4–97.9)	0.13	35.8 (2.9–1137.0)	0.99
ALT (U/L)^b^	29.0 (20.0–51.0)	25.5 (18.5–56.3)	0.73	40.3 (21.3–59.0)	0.23
AST (U/L)^b^	36.0 (26.0–52.3)	35.0 (22.0–55.7)	0.67	42.1 (25.0–80.0)	0.18
TB (µmol/L)^b^	12.5 (9.6–19.0)	16.1 (10.8–22.2)	0.20	12.7 (9.7–18.5)	0.70
CB (µmol/L)^b^	5.4 (4.0–8.1)	6.8 (4.6–11.0)	0.06	4.8 (3.5–9.8)	0.62
UCB (µmol/L)^b^	7.1 (5.1–10.9)	7.9 (5.8–13.6)	0.26	8.1 (5.4–11.3)	0.19
*D*_max_ (mm)^b^	49.0 (32.0–76.0)	39 (20.0–77.3)	0.17	43.0 (32.0–67.0)	0.40
Pathological subtype			0.51		0.83
HCC	118 (79.2%)	28 (73.7%)		30 (76.9%)	
ICC	31 (20.8%)	10 (26.3%)		9 (23.1%)	

Except where indicated, data are numbers of patients, with percentages in parentheses.

a Data are expressed as mean ± standard deviation.

b Data are medians, with interquartile range in parentheses.

p < 0.05 indicates there are significant differences in clinicopathological features of patients in the training set vs. test set and training set vs. validation set.

### Feature Extraction and Selection

From each patient, 1,409 features were extracted from the ultrasound images. Among the extracted features, fourteen 2D shape features were obtained only from the original images. Except for that, the features of all other six categories were obtained from one original image plus 14 derivative images. There were 18 first-order statistical features, 24 GLCM features, 16 GLRLM features, 16 GLSZM features, 14 GLDM features, and 5 NGTDM features. More information about the extracted features is listed in the **Supplementary Material 2**.

**Figure 4:**
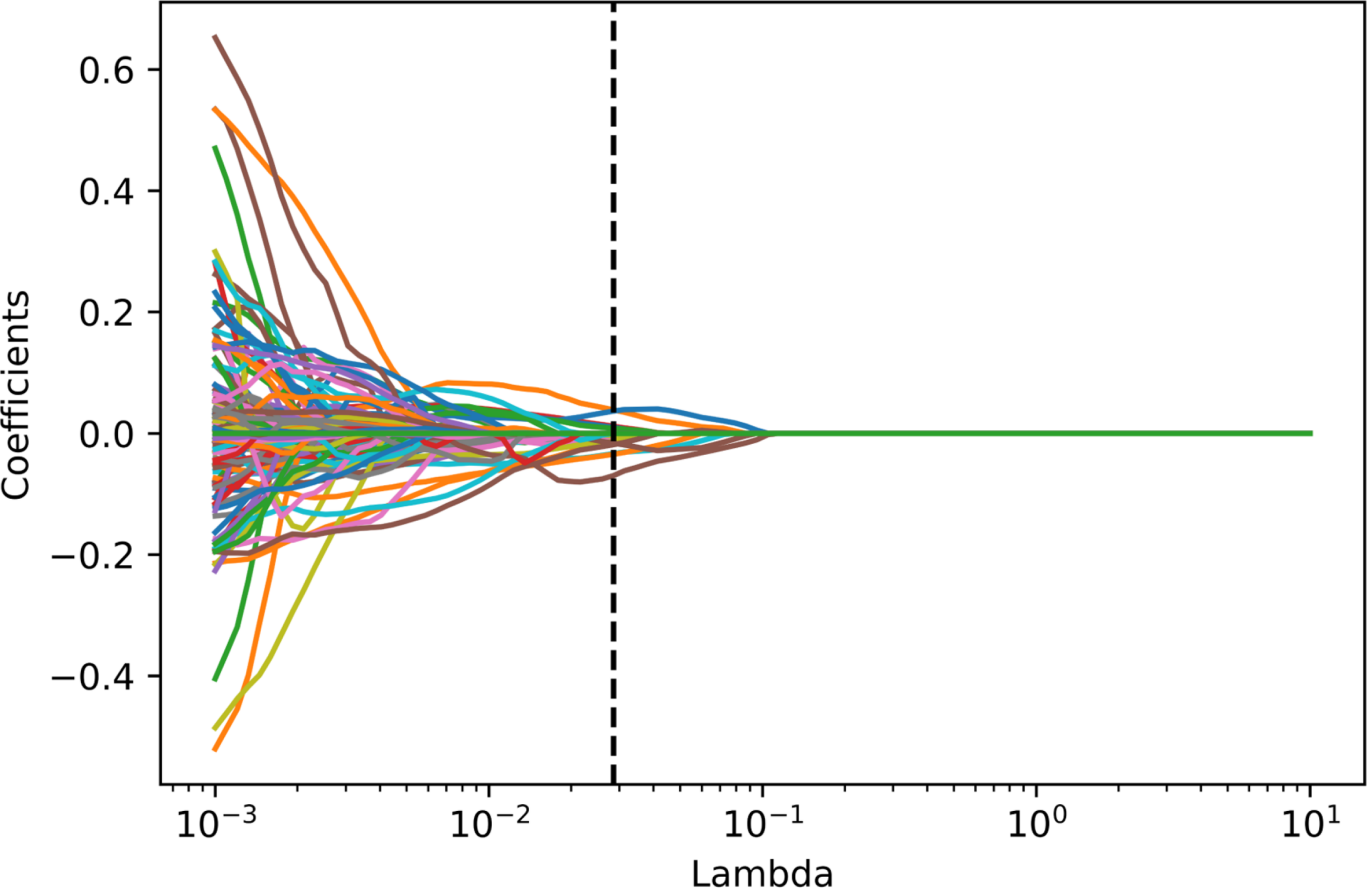
Radiomics feature selecting using the absolute shrinkage and selection operator (LASSO) regression model in the training dataset. In the LASSO model, the 10-fold cross-validation process was repeated 1,000,000 times to generate the optimal penalization coefficient lambda (*λ*). Finally, a *λ* value of 0.02848036 was chosen, which resulted in 14 nonzero coefficients.

Among these extracted features, 330 features with an intraclass correlation coefficient below 0.8 were first excluded. Then 16 features with zero variance were excluded using the variance filtering method. Lasso was used to reduce the dimensionality, which finally resulted in 14 features. The process of lasso feature selection is illustrated in **Figure 4**, with detailed information shown in the **Supplementary Figures S1–S3**.

### Predictive Performance of the Clinical Model and the Ultrasomics Model

**Figure 5:**
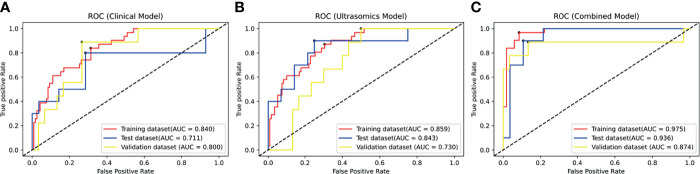
The ROC curves of the modes in the training dataset, test dataset, and validation dataset. **(A)** The ROC curve of the clinical model based on clinical factors. **(B)** The ROC curve of the radiomics model based on radiomics signature. **(C)** The ROC curve of the combined model based on clinical factors and radiomics signature.

The ROC curves of the clinical model and the ultrasomics model on the training set, test set, and independent validation set are shown in **Figures 5A, B**. On the test set, AUC (along with 95% CI), sensitivity, specificity, and accuracy of the clinical model and the ultrasomics model were 0.711 (0.541–0.846), 0.700, 0.714, and 0.711 vs. 0.843 (0.688–0.940), 0.900, 0.750, and 0.790, respectively. On the independent validation set, these performance indicators were 0.800 (0.641–0.911), 0.889, 0.667, and 0.718 vs. 0.730 (0.564–0.859), 0.667, 0.700, and 0.692, respectively. According to the results above, the ultrasomics-based model outperformed the clinical model in AUC and accuracy on the test set. The situation was just the opposite on the external validation set. The performance of the clinical model and the ultrasomics model in differentiation between HCC and ICC on the training set, test set, and independent validation set is shown in **Table 2**.

**Table 2:** Performance of training set, test set, and validation set.

Dataset	Model	Sensitivity (%)	Specificity (%)	Accuracy (%)	AUC (95%CI)	*p-*value
**Training set**	Clinical	77.42	68.64	70.47	0.840 (0.771–0.895)	<0.0001
	Ultrasomics	80.65	74.58	75.84	0.860 (0.793–0.911)	<0.0001
	Combined	96.77	87.29	89.26	0.975 (0.936–0.994)	<0.0001
**Test set**	Clinical	70.00	71.43	71.05	0.711 (0.541–0.846)	0.0757
	Ultrasomics	90.00	75.00	78.95	0.843 (0.688–0.940)	<0.0001
	Combined	90.00	85.71	86.84	0.936 (0.806–0.989)	<0.0001
**Validation set**	Clinical	88.87	66.67	71.79	0.800 (0.641–0.911)	0.0001
	Ultrasomics	66.67	70.00	69.23	0.730 (0.564–0.859)	0.0044
	Combined	88.87	86.67	87.18	0.874 (0.733–0.961)	<0.0001

p-value < 0.05 indicates a significant difference in the discrimination of HCC and ICC.

### Predictive Performance of the Combined Model

**Figure 5C** displays the ROC curves of the combined model on the training set, test set, and independent validation set. The AUC (along with 95% CI), sensitivity, specificity, and accuracy of the combined model on the test set and the independent validation set were 0.936 (0.806–0.989), 0.900, 0.857, and 0.868 *vs.* 0.874 (0.733–0.961), 0.889, 0.867, and 0.872 (**Table 2**), respectively. Thus, the combined model integrating ultrasomics signatures and clinical features had a better performance in differentiation between HCC and ICC than the other two models. The combined model had more stable performance and higher generalization ability (*p* < 0.05).

## Discussion

In clinical practice, physicians depend heavily on clinical symptoms, tumor serum markers, and imaging examination to differentiate between PLC subtypes before surgery. Since HCC and ICC share similar risk factors and clinical manifestations, the routine examination methods may lead to diagnostic mistakes. In the present study, ultrasomics signatures were generated using normalization, variance filtering, and lasso regression, and the prediction models were established by using SVM. The results showed that the ultrasomics signatures were successfully used to differentiate between HCC and ICC on the training set, test set, and the independent validation set (*p* < 0.05). The combined model outperformed the ultrasomics model on the test set, while the performance of clinical model was worse, the AUC of which was 0.936, 0.843, and 0.711, respectively. On the independent validation set, the performance of the combined model was still the best (*p* < 0.05). However, the performance of the ultrasomics model was worse than that of the clinical model (*p* < 0.05). The AUC was 0.874, 0.730, and 0.800, respectively. This was probably due to the differences in the type of equipment at diverse hospitals and different habits of using the ultrasound equipment among the physicians.

Medical imaging is an important diagnostic tool and plays an increasingly vital role as precision medicine continues to develop (40). It has been shown that imaging method based on multimodal imaging techniques can preoperatively differentiate between HCC and ICC to varying degrees. Ichikawa et al. determined the imaging hallmarks for distinguishing intrahepatic mass-forming biliary carcinomas (IMBCs) from HCC, and the diagnostic value was further verified by Bayesian statistics (AUC is 0.960) (41). However, only the radiographic manifestations of patients with good liver function and receiving surgical treatment were investigated, and such a selection bias might influence the results. Lewis et al. evaluated the ability of quantitative apparent diffusion coefficient (ADC) histogram analysis parameters and LI-RADS category in differentiating between HCC and other subtypes of PLC (42). In the two independent observers, the combined AUC of sex and LI-RADS and ADC at the fifth percentile for the diagnosis of liver cancer was 0.90 and 0.89, respectively. The result showed that HCC can be better distinguished from ICC and cHCC-ICC by combination of the ADC histogram parameters and LI-RADS categorization. However, there were a small number of samples and extracted features in their study. None of the studies above proceeded to deep mining and utilization of the radiographic images. As a result, a large number of tumor features and heterogeneity information of the tumor went unheeded.

As a branch of radiomics, ultrasomics has been proven helpful for liver fibrosis evaluation (43), differential diagnosis of liver tumors, and microvascular invasion assessment of HCC (44, 45). However, there have been few reports on the use of ultrasomics signature for the differentiation between HCC and ICC. Peng et al. applied ultrasomics analysis for noninvasive differentiation between the histopathological subtypes of PLC (33). The features were selected by using the Spearman correlation and lasso regression. Then the HCC-vs-non HCC radiomics model was constructed using a logistic regression algorithm. The AUC of which on the test set was 0.775. However, their findings were not subjected to multicenter validation. In our study, the AUC of the combined model for preoperative differentiation between HCC and ICC was 0.936 and 0.874 on the test set and the independent validation set, respectively, which were higher than those reported in the existing literature. It was indicated that ultrasomics seems to be potentially used clinically in the future.

However, there were also certain limitations in our study. Firstly, different grayscale ultrasound imaging systems were used to acquire the ultrasound images. Although the images were preprocessed before feature extraction, the use of different equipment for the imaging might affect the feature extraction results. Therefore, whether the established models are robust and universal remains to be further verified by incorporating more data. Secondly, all of the data were collected from consecutive cases in a retrospective manner, leading to inevitable selection bias. Therefore, it is necessary to increase the sample size in future studies. Moreover, the differentiation performance of the ultrasomics signatures and the established models remains to be further verified by a prospective study. Thirdly, only two subtypes of PLC, namely, HCC and ICC, were covered in our study, but the rare subtypes, such as the mixed liver cancer, were not. The data of other subtypes of liver cancer should be included in future studies for optimized universality and clinical value of the models.

Taken together, the clinical value of machine learning-based ultrasomics was confirmed for the preoperative noninvasive differentiation between HCC and ICC. The combined model not only had a better performance in differentiation between HCC and ICC than either the clinical model or the ultrasomics model alone but also had a higher generalization ability.

## Data Availability Statement

The original contributions presented in the study are included in the article/**Supplementary Material**; further inquiries can be directed to the corresponding author.

## Ethics Statement

The study involving human participants were reviewed and approved by the ethics committees, and the written informed consents were waived.

## Author Contributions

LZ and SR conceived and designed the study. SR, QL, QQ, SD, BM, XL, and YW collected the data. LZ, SR, and SL analyzed the data. SR and LZ wrote the paper. All authors contributed to the article and approved the submitted version.

## Funding

This study has received funding by the National Key Research and Development Program of China (Grant No. 2018YFC0114606).

## Conflict of Interest

The authors declare that the research was conducted in the absence of any commercial or financial relationships that could be construed as a potential conflict of interest.

## Publisher’s Note

All claims expressed in this article are solely those of the authors and do not necessarily represent those of their affiliated organizations, or those of the publisher, the editors and the reviewers. Any product that may be evaluated in this article, or claim that may be made by its manufacturer, is not guaranteed or endorsed by the publisher.

## Abbreviations

PLC, primary liver cancer; HCC, hepatocellular carcinoma; ICC, intrahepatic cholangiocarcinoma; AFP, alpha-fetoprotein; ALT, alanine aminotransferase; AST, aspartate aminotransferase; TB, total bilirubin; CB, conjugated bilirubin; UCB, unconjugated bilirubin; DICOM, digital imaging and communications in medicine; GLCM, gray-level cooccurrence matrix; GLDM, gray-level dependence matrix; GLRLM, gray-level run length matrix; GLSZM, gray-level size-zone matrix; NGTDM, neighboring gray tone difference matrix; ROI, region of interest; AUC, area under the curve; ROC, receiver operating characteristic; LASSO, least absolute shrinkage and selection operator; SVM, support vector machine

## References

[1] Njei B Rotman Y Ditah I Lim JK. Emerging Trends in Hepatocellular Carcinoma Incidence and Mortality. Hepatology (2015) 61(1):191–9. doi: 10.1002/hep.27388 PMC4823645 25142309

[2] L SR D MK E FH Ahmedin J. Cancer Statistics. CA Cancer J Clin (2021) 71(1):7–33. doi: 10.3322/CAAC.21654 33433946

[3] LJ M Kate KR Augusto V SA G Eli P Sasan R. Hepatocellular Carcinoma. Nat Rev Dis Primers (2021) 7(1):6. doi: 10.1038/S41572-020-00240-3 33479224 PMC13537433

[4] Nagtegaal ID Odze RD Klimstra D Paradis V Rugge M Schirmacher P. The 2019 WHO Classification of Tumours of the Digestive System. Histopathology (2020) 76(2):182–8. doi: 10.1111/his.13975 PMC7003895 31433515

[5] Sia D Villanueva A Friedman SL Llovet JM. Liver Cancer Cell of Origin, Molecular Class, and Effects on Patient Prognosis. Gastroenterology (2017) 152(4):745–61. doi: 10.1053/j.gastro.2016.11.048 PMC12160040 28043904

[6] Veronika B Vita Z Roman H Pavel F Jiri N Marketa H. Differential Expression of Anterior Gradient Protein 3 in Intrahepatic Cholangiocarcinoma and Hepatocellular Carcinoma. Exp Mol Pathol (2014) 96(3):375–81. doi: 10.1016/j.yexmp.2014.04.002 24747240

[7] Bruix J Reig M Sherman M. Evidence-Based Diagnosis, Staging, and Treatment of Patients With Hepatocellular Carcinoma. Gastroenterology (2016) 150(4):835–53. doi: 10.1053/j.gastro.2015.12.041 26795574

[8] Gaya S Yuhree K Sorin A Irinel P P MH Luca A. Is Hepatic Resection for Large or Multifocal Intrahepatic Cholangiocarcinoma Justified? Results From a Multi-Institutional Collaboration. Ann Surg Oncol (2015) 22(7):2218–25. doi: 10.1245/s10434-014-4223-3 PMC4834710 25354576

[9] Banales JM Iñarrairaegui M Arbelaiz A Milkiewicz P Muntané J Muñoz-Bellvis L. Serum Metabolites as Diagnostic Biomarkers for Cholangiocarcinoma, Hepatocellular Carcinoma, and Primary Sclerosing Cholangitis. Hepatology (2019) 70(2):547–62. doi: 10.1002/hep.30319 PMC6767196 30325540

[10] Kate KR John B J GG X ZA. Systemic Therapies for Intrahepatic Cholangiocarcinoma. J Hepatol (2020) 72(2):353–63. doi: 10.1016/j.jhep.2019.10.009 31954497

[11] Tong-Chun X Bo-Heng Z Sheng-Long Y Zheng-Gang R. Differentially Expressed Gene Profiles of Intrahepatic Cholangiocarcinoma, Hepatocellular Carcinoma, and Combined Hepatocellular-Cholangiocarcinoma by Integrated Microarray Analysis. Tumour Biol J Int Soc Oncodevelopmental Biol Med (2015) 36(8):5891–9. doi: 10.1007/s13277-015-3261-1 25712376

[12] Jittiporn C Anuradha B Hien D Siritida R Benjarath P Mee KS. Common Molecular Subtypes Among Asian Hepatocellular Carcinoma and Cholangiocarcinoma. Cancer Cell (2017) 32(1):57–70.e3. doi: 10.1016/j.ccell.2017.05.009 28648284 PMC5524207

[13] Chu H Liu Z Liang W Zhou Q Zhang Y Lei K. Radiomics Using CT Images for Preoperative Prediction of Futile Resection in Intrahepatic Cholangiocarcinoma. Eur Radiol (2020) 31(4):2368–76. doi: 10.1007/s00330-020-07250-5 33033863

[14] Bin H Lu W Xin-Yuan L Feng X Cai-Feng L Wei-Feng S. Small Intrahepatic Cholangiocarcinoma and Hepatocellular Carcinoma in Cirrhotic Livers May Share Similar Enhancement Patterns at Multiphase Dynamic MR Imaging. Radiology (2016) 281(1):150–7. doi: 10.1148/radiol.2016151205 27077381

[15] Forner A Llovet JM Bruix J. Hepatocellular Carcinoma. Lancet (2012) 379(9822):1245–55. doi: 10.1016/S0140-6736(11)61347-0 PMC13537732 22353262

[16] Rahnemai-Azar AA Weisbrod A Dillhoff M Schmidt C Pawlik TM. Intrahepatic Cholangiocarcinoma: Molecular Markers for Diagnosis and Prognosis. Surg Oncol (2017) 26(2):125–37. doi: 10.1016/j.suronc.2016.12.009 28577718

[17] Losic B Craig AJ Villacorta-Martin C Martins-Filho SN Akers N Chen X. Intratumoral Heterogeneity and Clonal Evolution in Liver Cancer. Nat Commun (2020) 11(5):1450–62. doi: 10.1038/s41467-019-14050-z PMC6962317 31941899

[18] Wei J Jiang H Gu D Niu M Fu F Han Y. Radiomics in Liver Diseases: Current Progress and Future Opportunities. Liver Int (2020) 40(9):2050–63. doi: 10.1111/liv.14555 PMC7496410 32515148

[19] Wu M Tan H Gao F Hai J Ning P Chen J. Predicting the Grade of Hepatocellular Carcinoma Based on Non-Contrast-Enhanced MRI Radiomics Signature. Eur Radiol (2019) 29(6):2802–11. doi: 10.1007/s00330-018-5787-2 30406313

[20] Zhou W Zhang L Wang K Chen S Wang G Liu Z. Malignancy Characterization of Hepatocellular Carcinomas Based on Texture Analysis of Contrast-Enhanced MR Images. J Magn Reson Imaging (2017) 45(5):1476–84. doi: 10.1002/jmri.25454 27626270

[21] Ren S Qi Q Liu S Duan S Zhang L Mao B. Preoperative Prediction of Pathological Grading of Hepatocellular Carcinoma Using Machine Learning-Based Ultrasomics: A Multicenter Study. Eur J Radiol (2021) 143:109891. doi: 10.1016/j.ejrad.2021.109891 34481117

[22] Wang W Wu SS Zhang JC Xian MF Huang H Li W. Preoperative Pathological Grading of Hepatocellular Carcinoma Using Ultrasomics of Contrast-Enhanced Ultrasound. Acad Radiol (2021) 28(8):1094–101. doi: 10.1016/j.acra.2020.05.033 32622746

[23] Rodrigo C Farhad M Manuel P Avinash K Dushyant S. Characterization of Portal Vein Thrombosis (Neoplastic Versus Bland) on CT Images Using Software-Based Texture Analysis and Thrombus Density (Hounsfield Units). Am J Roentgenology (2016) 207(5):W81–7. doi: 10.2214/AJR.15.15928 27490095

[24] Shaimaa B Sebastian E Rajesh S Aya K John L Sandy N. Noninvasive Radiomics Signature Based on Quantitative Analysis of Computed Tomography Images as a Surrogate for Microvascular Invasion in Hepatocellular Carcinoma: A Pilot Study. J Med Imaging (2017) 4(4):41303. doi: 10.1117/1.JMI.4.4.041303 PMC5565686 28840174

[25] Suh S-W Lee K-W Lee J-M You T YoungRok C Hyeyoung K. Prediction of Aggressiveness in Early-Stage Hepatocellular Carcinoma for Selection of Surgical Resection. J Hepatol (2014) 60(6):1219–24. doi: 10.1016/j.jhep.2014.01.027 24548529

[26] Sébastien M Gérard T Charlotte C Carole D Alain R Alain L. Advanced Hepatocellular Carcinoma: Pretreatment Contrast-Enhanced CT Texture Parameters as Predictive Biomarkers of Survival in Patients Treated With Sorafenib. Radiology (2018) 288(2):445–55. doi: 10.1148/radiol.2018171320 29584597

[27] Kim TH Kim SY Tang A Lee JM. Comparison of International Guidelines for Noninvasive Diagnosis of Hepatocellular Carcinoma: 2018 Update. Clin Mol Hepatol (2019) 25(3):245–63. doi: 10.3350/cmh.2018.0090 PMC6759428 30759967

[28] Yilmaz N Yilmaz UE Suer K Goral V Cakir N. Screening for Hepatocellular Carcinoma: Summary of Current Guidelines Up to 2018. Hepatoma Res (2018) 4(8):64–73. doi: 10.20517/2394-5079.2018.49

[29] Luo W Huang Q Huang X Hu H Zeng F Wang W. Predicting Breast Cancer in Breast Imaging Reporting and Data System (BI-RADS) Ultrasound Category 4 or 5 Lesions: A Nomogram Combining Radiomics and BI-RADS. Sci Rep (2019) 9(3):889–94. doi: 10.1038/s41598-019-48488-4 PMC6695380 31417138

[30] Park VY Han K Lee E Kim E-K Moon HJ Yoon JH. Association Between Radiomics Signature and Disease-Free Survival in Conventional Papillary Thyroid Carcinoma. Sci Rep (2019) 9(Suppl 1):1535–40. doi: 10.1038/s41598-018-37748-4 PMC6418281 30872763

[31] Chen LD Li W Xian MF Zheng X Wang W. Preoperative Prediction of Tumour Deposits in Rectal Cancer by an Artificial Neural Network–Based US Radiomics Model. Eur Radiol (2020) 30(4):1969–79. doi: 10.1007/s00330-019-06558-1 31828415

[32] Sun P Wang D Mok V Shi L. Comparison of Feature Selection Methods and Machine Learning Classifiers for Radiomics Analysis in Glioma Grading. IEEE Access (2019) 7:102010–20. doi: 10.1109/ACCESS.2019.2928975

[33] Yuting P Peng L Linyong W Da W Yujia Z Li L. Ultrasound-Based Radiomics Analysis for Preoperatively Predicting Different Histopathological Subtypes of Primary Liver Cancer. Front Oncol (2020) 10:1646. doi: 10.3389/fonc.2020.01646 33072550 PMC7543652

[34] Yushkevich PA Piven J Hazlett HC Smith RG Ho S Gee JC. User-Guided 3D Active Contour Segmentation of Anatomical Structures: Significantly Improved Efficiency and Reliability. Neuroimage (2006) 31(3):1116–28. doi: 10.1016/j.neuroimage.2006.01.015 16545965

[35] Leijenaar RTH Nalbantov G Carvalho S Elmpt W Troost EGC Boellaard R. The Effect of SUV Discretization in Quantitative FDG-PET Radiomics: The Need for Standardized Methodology in Tumor Texture Analysis. Sci Rep (2015) 5(1):27–40. doi: 10.1038/srep11075 PMC4525145 26242464

[36] Alex Z M.A A A A Saeed A J B Marta B. PO-0981: Results From the Image Biomarker Standardisation Initiative. Radiother Oncol (2018) 127:S543–4. doi: 10.1016/S0167-8140(18)31291-X

[37] Géron A. Hands-On Machine Learning With Scikit-Learn and TensorFlow: Concepts, Tools, and Techniques to Build Intelligent Systems. O’Reilly Beijing (2017).

[38] DE R DD M L C-PD. Comparing the Areas Under Two or More Correlated Receiver Operating Characteristic Curves: A Nonparametric Approach. Biometrics (1988) 44(3):837–45. doi: 10.2307/2531595 3203132

[39] Koo TK Li MY. A Guideline of Selecting and Reporting Intraclass Correlation Coefficients for Reliability Research. J Chiropr Med (2016) 15(2):155–63. doi: 10.1016/j.jcm.2016.02.012 PMC4913118 27330520

[40] Saini A Breen I Pershad Y Naidu S Knuttinen MG Alzubaidi S. Radiogenomics and Radiomics in Liver Cancers. Diagnostics (2018) 9(1):4. doi: 10.3390/diagnostics9010004 PMC6468592 30591628

[41] Shintaro I Hiroyoshi I Tatsuya S Daiki T Kojiro T Kaori T. Distinguishing Intrahepatic Mass-Forming Biliary Carcinomas From Hepatocellular Carcinoma by Computed Tomography and Magnetic Resonance Imaging Using the Bayesian Method: A Bi-Center Study. Eur Radiol (2020) 30(11):5992–6002. doi: 10.1007/s00330-020-06972-w 32500195

[42] Lewis S Peti S Hectors SJ King M Rosen A Kamath A. Volumetric Quantitative Histogram Analysis Using Diffusion-Weighted Magnetic Resonance Imaging to Differentiate HCC From Other Primary Liver Cancers. Abdominal Radiol (2019) 44(3):912–22. doi: 10.1007/s00261-019-01906-7 30712136

[43] Li W Huang Y Zhuang B Liu G Hu H Li X. Multiparametric Ultrasomics of Significant Liver Fibrosis: A Machine Learning-Based Analysis. Eur Radiol (2019) 29(3):1496–506. doi: 10.1007/s00330-018-5680-z PMC6510867 30178143

[44] Hu H Wang Z Huang X Chen S Zheng X Ruan S. Ultrasound-Based Radiomics Score: A Potential Biomarker for the Prediction of Microvascular Invasion in Hepatocellular Carcinoma. Eur Radiol (2019) 29(6):2890–901. doi: 10.1007/s00330-018-5797-0 30421015

[45] Mao B Ma J Duan S Xia Y Tao Y Zhang L. Preoperative Classification of Primary and Metastatic Liver Cancer via Machine Learning-Based Ultrasound Radiomics. Eur Radiol (2021) 31(7):4576–86. doi: 10.1007/s00330-021-07704-4 33447862

