# Supplementary material for: Clinical Value of Machine Learning-Based Ultrasomics in Preoperative Differentiation Between Hepatocellular Carcinoma and Intrahepatic Cholangiocarcinoma: A Multicenter Study
Source: Front Oncol. 2021 Nov 5;11:749137. doi: 10.3389/fonc.2021.749137 (PMC8604281; doi:10.3389/fonc.2021.749137)
Supplement: Supplementary file 1 [file DataSheet_1.docx]

Supplementary Material

# Supplementary 1: Feature extraction

In this study, radiomics features of each patient were extracted from both original images and derived images which were obtained by applying 14 filters to the original image, such as Wavelet (wavelet-LLH, wavelet-LHL, wavelet-LHH, wavelet-HLH, wavelet-HLL, wavelet-LLL, wavelet-HHL, wavelet-HHH), Square, SquareRoot, Logarithm, Exponential, Gradient, LocalBinaryPattern2D, using Pyradiomics v.2.1.2 package.

# Supplementary 2: Feature selection

T-test and Lasso regression algorithm were used for feature selection, and 14 features were finally obtained, which were listed in the following table:

| No. | Feature Name | LASSO Coefficient |
| --- | --- | --- |
| 1 | wavelet-LHL_glszm_SizeZoneNonUniformityNormalized | -0.033490 |
| 2 | wavelet-LHH_glcm_Imc2 | 0.011652 |
| 3 | wavelet-LHH_glszm_ZoneEntropy | 0.038709 |
| 4 | wavelet-HLH_firstorder_Minimum | -0.005606 |
| 5 | wavelet-HLH_glcm_ClusterShade | -0.034641 |
| 6 | wavelet-HHL_glcm_ClusterShade | 0.009771 |
| 7 | wavelet-HHL_glrlm_RunVariance | 0.036573 |
| 8 | wavelet-LLL_glrlm_LongRunEmphasis | -0.003584 |
| 9 | wavelet-LLL_glrlm_LongRunLowGrayLevelEmphasis | -0.013667 |
| 10 | wavelet-LLL_glszm_LargeAreaEmphasis | -0.003567 |
| 11 | wavelet-LLL_ngtdm_Busyness | 0.008272 |
| 12 | square_firstorder_RootMeanSquared | -0.016189 |
| 13 | logarithm_firstorder_Kurtosis | -0.018773 |
| 14 | gradient_firstorder_90Percentile | -0.069924 |

# Supplementary 3: Model training

In the training of clinical model, radiomics model and combined model, radial basis function (RBF) was firstly selected using learning curve. Then, grid search was used to select the optimal combination of parameter for kernel coefficient λ, penalty factor *C* and sample weight parameter *class_weight*.

# Supplementary 4: Model construction

For the clinical model, it is necessary to first encode the text data for conversion into numerical data, for example: Gender: "male" is coded as "0" and "female" is coded as "1"; Hepatitis history: "None" is coded as "0" and "yes" is coded as "1". Then, the numerical data (including gender, age, history of hepatitis, AFP, ALT, AST, TB, CB, UCB and the size of lesion) were Z-score normalized to eliminate the problem of different value scales caused by different units and value ranges of various clinical data. Finally, these 10 clinical features were used for clinical model training and evaluation.

For the ultrasomics model, first Z-score normalization was used to preprocess the extracted 1,409 features, and then a series of methods such as intraclass correlation, coefficient variance and filtering lasso method were used for feature selection. Finally, the obtained 14 ultrasomics signatures were used for model training and evaluation.

For the combined model, the 10 clinical features of each patient obtained after pretreatment and the corresponding 14 ultrasonic omics features were combined, and then the 24 features were used for model training and evaluation.

# Supplementary Figures


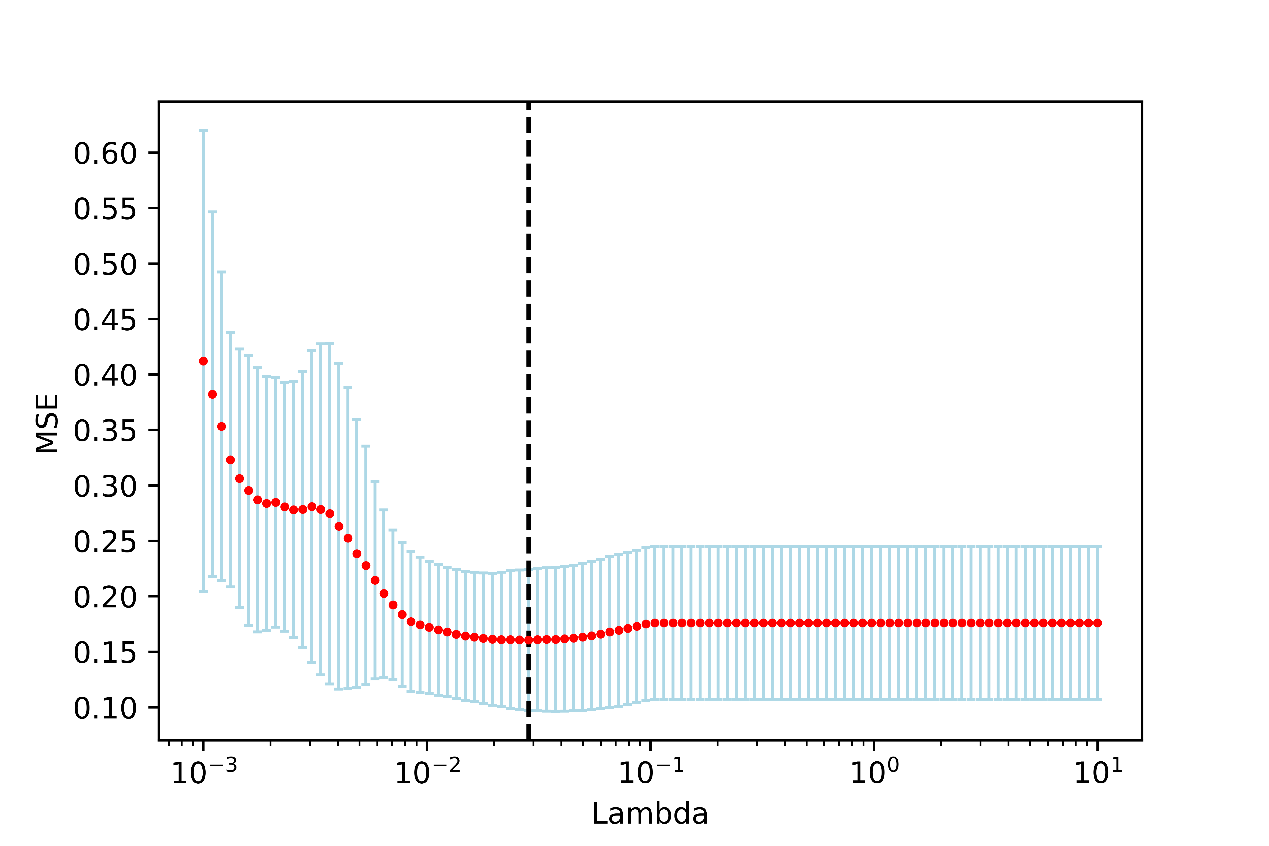


**Supplementary Figure 1.** The LASSO path of feature selection using lasso regression algorithm.


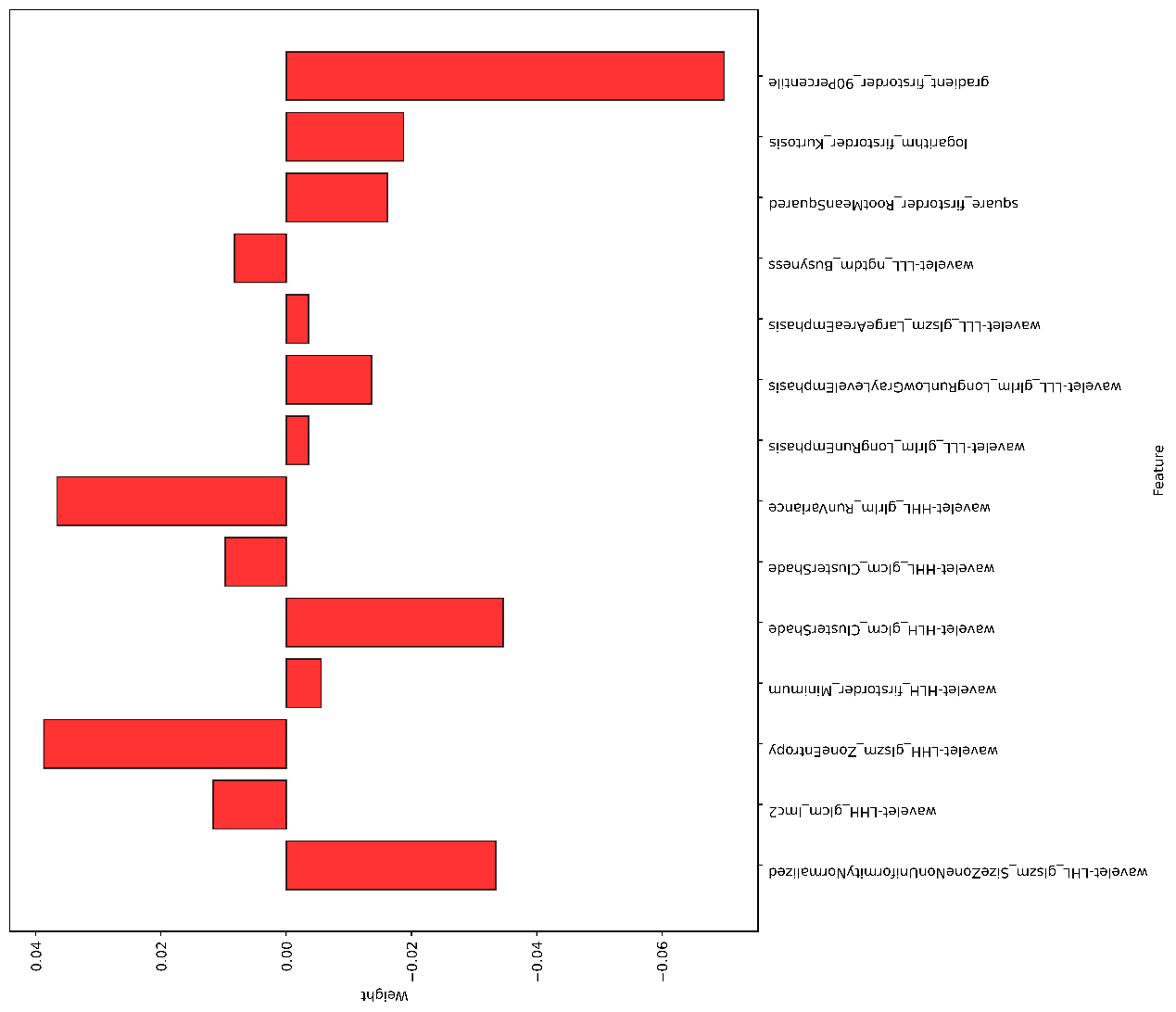


**Supplementary Figure 2.** The LASSO coefficient of each radiomics signatures.


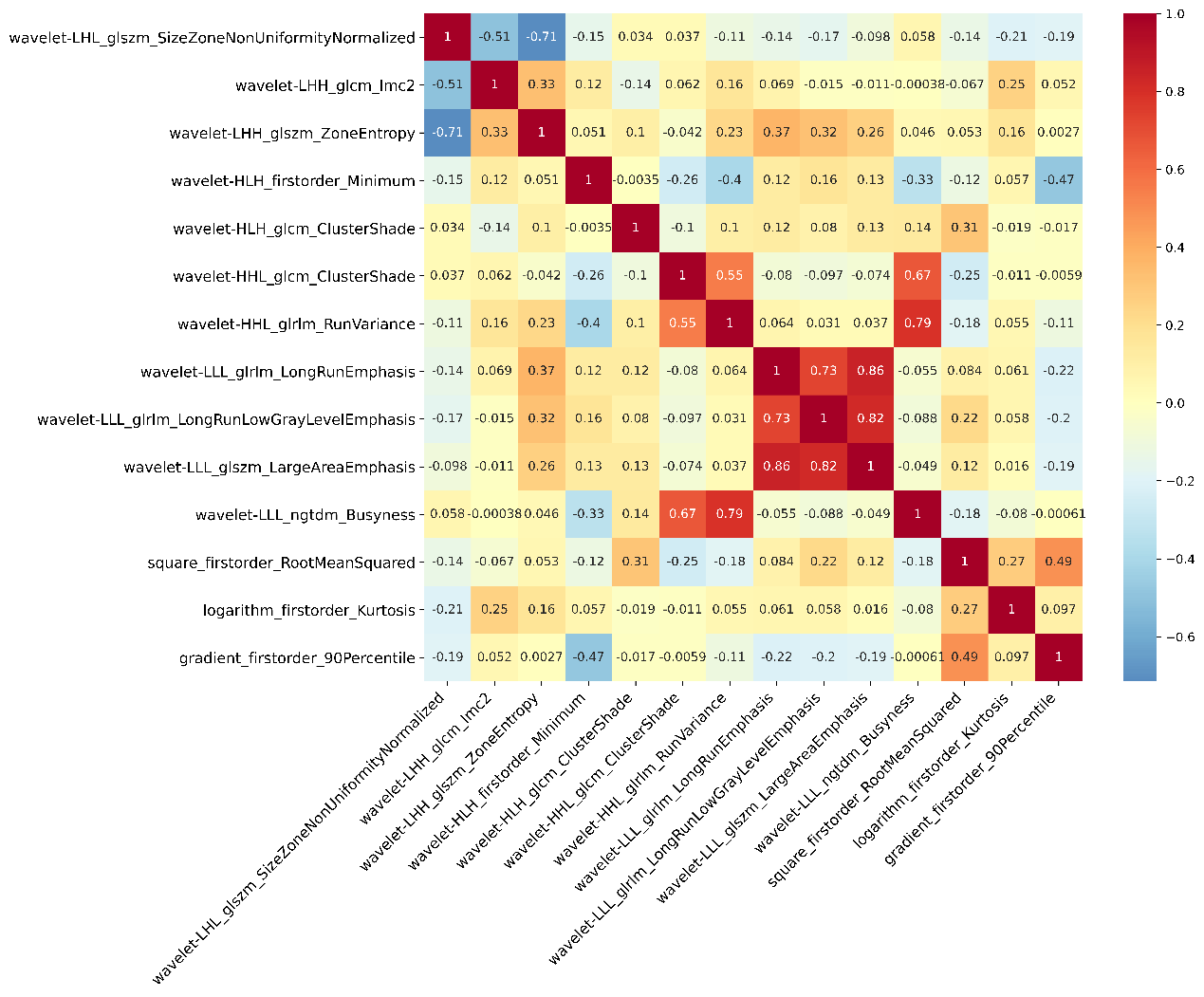


**Supplementary Figure 3.** The heatmap of correlation coefficient of radiomics signatures.

# Supplementary Tables

**Supplementary Table 1.** Performance of training set, test set and validation set

| Dataset | Model | Sensitivity  (%) | Specificity  (%) | Accuracy  (%) | AUC  (95%CI) | PPV  (%) | | NPV  (%) |
| --- | --- | --- | --- | --- | --- | --- | --- | --- |
| Training set | Clinical | 77.42 | 68.64 | 70.47 | 0.840(0.771-0.895) | 39.34 | 92.05 | |
|  | Ultrasomics | 80.65 | 74.58 | 75.84 | 0.860(0.793-0.911) | 45.45 | 93.62 | |
|  | Combined | 96.77 | 87.29 | 89.26 | 0.975(0.936-0.994) | 66.67 | 99.04 | |
| Test set | Clinical | 70.00 | 71.43 | 71.05 | 0.711(0.541-0.846) | 46.67 | 86.96 | |
|  | Ultrasomics | 90.00 | 75.00 | 78.95 | 0.843(0.688-0.940) | 56.25 | 95.45 | |
|  | Combined | 90.00 | 85.71 | 86.84 | 0.936(0.806-0.989) | 69.23 | 96.00 | |
| Validation set | Clinical | 88.87 | 66.67 | 71.79 | 0.800(0.641-0.911) | 44.44 | 95.24 | |
|  | Ultrasomics | 66.67 | 70.00 | 69.23 | 0.730(0.564-0.859) | 40.00 | 87.50 | |
|  | Combined | 88.87 | 86.67 | 87.18 | 0.874(0.733-0.961) | 66.67 | 96.30 | |
